# Adaptation of a Trauma‐Focused Parenting Intervention for Military‐Connected Mothers and Their Young Children: A Rapid Qualitative Study

**DOI:** 10.1111/famp.70209

**Published:** 2026-10-19

**Authors:** Kathrine S. Sullivan, Gina M. Angelotti, Seonyeong Kim, Whitney K. Wortham, Fan Zhang, Stephanie Campos, Damaris Banegas, Michael A. Lindsey, Susan G. Timmer, Marylene Cloitre, Christie Jackson, James Jaccard, Melissa E. Smith, Alison B. Hamilton, David J. Linkh, Steven L. Lancaster

**Affiliations:** ^1^ Silver School of Social Work New York University New York New York USA; ^2^ Graduate School of Arts and Science New York University New York New York USA; ^3^ CAARE Diagnostic & Treatment Center, Department of Pediatrics University of California Sacramento California USA; ^4^ STAIR Institute New York New York USA; ^5^ School of Social Work University of Maryland Baltimore Maryland USA; ^6^ Department of Psychiatry and Biobehavioral Sciences University of California Los Angeles Los Angeles California USA; ^7^ Cohen Veterans Network Institute for Quality Stamford Connecticut USA

**Keywords:** adaptation, evidence‐based interventions, military family, rapid qualitative analysis, trauma; parenting

## Abstract

Adapting evidence‐based interventions (EBIs) to be sensitive to populations who have experienced trauma has been shown to improve engagement, retention, satisfaction, and effectiveness. Compared to civilian mothers, military‐connected mothers (MCM)—including service members, veterans, or their partners—face greater exposure to trauma across the life course, leading to mental health and parenting challenges. These may adversely affect their children's mental health and perpetuate generational trauma. EBIs aligned with people's life experiences and cultural backgrounds are critical to supporting the mental wellbeing and parental functioning of MCM. Informed by the Framework for Reporting Adaptations and Modifications‐Enhanced and utilizing rapid qualitative analysis—a rigorous method for quickly identifying key interview themes—this paper describes the process of adapting an innovative EBI, Parenting‐STAIR (P‐STAIR), which targets maternal trauma, emotion regulation, and parenting. Transcripts from interviews with administrators, clinicians, and MCM at clinics serving military families were systematically summarized, organized into matrices, and analyzed. Four emergent themes informed adaptations to P‐STAIR: (a) streamlined treatment and enhanced engagement, (b) flexibility and responsiveness, (c) mental health stigma reduction, and (d) military cultural relevance. Considering these priorities, which were expressed across participant groups, P‐STAIR was shortened from 23 sessions to a brief, modular 12–16‐session intervention that can be personalized to individual needs and uses approachable language (e.g., distress vs. trauma), which aims to reduce the impact of stigma on engagement and retention. A standalone guide was developed to accompany the intervention manual, which may help clinicians better understand the unique experiences of military families. The outcome of this process, PSTAIR‐M, is a briefer, modular intervention we anticipate will be suitable for delivery to MCM.

## 1 Introduction

The mental health impacts of military service extend beyond service members to their families, influencing various aspects of well‐being, family dynamics, and mental health (National Academies of Sciences, Engineering, and Medicine [NASEM] 2019). As research on military families has expanded, greater attention has been given to military‐connected mothers (MCM), a population that includes service members, veterans, and spouses of service members or veterans. MCM experience distinct challenges that differentiate them from civilian mothers (NASEM 2019; Sullivan et al. 2020). These challenges include frequent relocations, prolonged separations due to deployments, disruptions to spousal employment, and increased social isolation resulting from military‐related mobility (NASEM 2019). Furthermore, MCM are embedded within a military culture that emphasizes hierarchy, cohesion, and resilience. This contributes to stigma surrounding mental health treatment and hinders engagement in care (Atuel and Castro 2018; Coll et al. 2011). These cultural factors highlight the need for interventions that are tailored to the specific experiences of MCM and the contextual barriers they face. Guided by the Framework for Reporting Adaptations and Modifications‐Enhanced (FRAME) approach (Wiltsey Stirman et al. 2019), the purpose of this paper is to describe the process of adapting a specific evidence‐based intervention (EBI), Parenting‐STAIR, to meet the needs of MCM.

### 1.1 Trauma Exposure Among MCM and Its Impact on Parenting

MCM experience disproportionately high rates of trauma exposure, which places them at heightened risk for mental health challenges (Lehavot et al. 2018; O'Grady et al. 2016; Zinzow et al. 2007). Notably, while military‐related stressors have been linked to adverse psychological outcomes among MCM (Institute of Medicine 2010; NASEM 2019), emerging evidence suggests that these stressors alone do not fully explain the mental health difficulties experienced by MCM. Childhood adversity is more prevalent among MCM than their civilian counterparts, and these early‐life traumas have a substantial influence on later mental health (Blosnich et al. 2014; Lehavot et al. 2018; O'Grady et al. 2016). Recent findings underscore that childhood adversity is the strongest predictor of negative mental health outcomes among military‐connected parents and may exacerbate the impact of other stressors (Sullivan, Park, et al. 2023). The cumulative burden of both early and recent stressors among military parents is consequential for parenting and child outcomes (Sullivan et al. 2024).

A robust literature has established that maternal posttraumatic stress disorder (PTSD) and depression are associated with compromised parenting practices, including decreased sensitivity, heightened stress, and greater use of maladaptive disciplinary strategies (Creech and Misca 2017; Sherman et al. 2016). Moreover, evidence of bidirectionality suggests that these parenting challenges can, in turn, exacerbate maternal mental health symptoms (Creech and Misca 2017; Janke‐Stedronsky et al. 2016; Sherman et al. 2016). Such reciprocal influences place children at elevated risk of developing mental health problems (Cooke et al. 2019; Lambert et al. 2014) and perpetuate the intergenerational transmission of trauma (Madigan et al. 2019).

### 1.2 The Need for a Trauma‐Informed Parenting Intervention for MCM

Though effective, existing parenting programs for military families are limited in scope, as they primarily focus on deployment‐related stressors, do not directly treat parental trauma, are delivered only to parents without directly involving children, and/or are offered in less flexible multifamily formats that inhibit tailoring to individual family needs (Cheng et al. 2024; Gewirtz et al. 2014, 2018; Liu et al. 2024; Rosenblum and Muzik 2014). These constraints leave critical gaps in addressing the broader impact of childhood adversity and in providing personalized, skill‐based coaching that directly enhances parenting outcomes (Shanley and Niec 2010; Sullivan, Park, et al. 2023). Recognizing these gaps, there is a clear need for parenting interventions that address maternal trauma‐related mental health and are culturally adapted to the specific needs of MCM. Such interventions should comprehensively address trauma exposure across the life course, integrate parenting‐focused components, and incorporate military cultural considerations to enhance engagement and retention.

### 1.3 Parenting‐STAIR

Parenting‐STAIR (P‐STAIR) integrates two well‐established EBIs: Skills Training in Affective and Interpersonal Regulation (STAIR) and Parent–Child Care (PC‐CARE). STAIR is an EBI that incorporates emotion regulation skill‐building and narrative exposure to reduce PTSD symptoms and improve social functioning (Cloitre et al. 2002; Jain et al. 2020). PC‐CARE is a dyadic intervention that enhances positive parenting practices, strengthens parent–child relationships, and improves child behavioral outcomes (Hawk et al. 2020, 2024; Timmer et al. 2019). By combining these two interventions, P‐STAIR simultaneously addresses maternal trauma symptoms and parenting skills, ensuring that both maternal mental health and parent–child interactions are targeted in treatment. Prior trials of P‐STAIR demonstrate strong feasibility and acceptability, with high engagement among vulnerable families experiencing significant instability. Mothers demonstrated substantial improvements in emotion regulation, PTSD, depression, and parenting stress, alongside better parenting skills and reductions in child behavior problems and maltreatment risk (Sullivan, Ancharski, et al. 2023; Wortham et al. 2024). P‐STAIR is ideally suited for adaptation to meet the needs of MCM because it addresses trauma across the lifecourse, gives attention to maternal mental health, and provides individualized parent coaching to mothers and their young children (Sullivan, Ancharski, et al. 2023).

### 1.4 The Current Paper

The purpose of the current paper is to describe the systematic process employed to adapt P‐STAIR to be both culturally appropriate for MCM and contextually appropriate for the setting in which the intervention will be delivered. Adaptation of EBIs is a fundamental aspect of implementation science, facilitating the alignment of interventions with the needs of target populations while maintaining fidelity to core treatment components (Wiltsey Stirman et al. 2019). Modifications that enhance the cultural and contextual fit of EBIs have been shown to improve engagement, acceptability, and clinical effectiveness, particularly among historically underserved or high‐risk populations (Rathod et al. 2018; Wiltsey Stirman et al. 2019). This study thus advances the field by adapting and evaluating a trauma‐informed parenting intervention for MCM within a military cultural context while leveraging a rigorous adaptation framework to guide implementation and evaluation. Findings from this work will provide critical insights into how interventions can be tailored to address the needs of trauma‐exposed mothers and their children in military families while preserving fidelity and maximizing effectiveness.

## 2 Method

### 2.1 Setting

This study was conducted in three community mental health clinics, which are part of Cohen Veterans Network (CVN). CVN is a network of 22 community‐based clinics in 15 states, which are operated by partner organizations but follow a standardized model. CVN was launched in 2016 with a philanthropic gift from Steven A. Cohen with the goal of serving post‐9/11 service members, veterans and their families. As such, CVN clinics are an ideal venue to reach MCM, the target population for the present study, as this population may not be adequately served at Defense Health Agency or Veterans Affairs clinics. Three clinics were initially selected as sites for a planned pilot randomized control trial of the adapted P‐STAIR because they offered access to a diverse population of military‐connected families and an adequate flow of potential participants to support the trial's recruitment targets. For qualitative inquiry to inform the adaptation of P‐STAIR, recruitment of clinicians and MCM was initially planned to occur at these three clinics as well. However, as described below, recruitment of MCM was slower than anticipated, so ultimately recruitment was expanded to the entire network.

### 2.2 Structured Approach to Intervention Adaptation

To ensure transparency and rigor in the adaptation process, we employ FRAME (Wiltsey Stirman et al. 2019). FRAME provides a structured method for documenting intervention modifications by capturing key dimensions of adaptation, including the timing of modifications, whether adaptations are planned or reactive, and the rationale for modifications. By systematically applying FRAME, we aimed to ensure that the adapted P‐STAIR remains evidence‐based while maximizing fit for the target population. In particular, FRAME was applied in this context to support our consideration of the cultural needs of MCM as well as the clinical context in which P‐STAIR will be delivered. This structured reporting approach not only enhances the reproducibility of our adaptation process but also contributes to broader implementation science efforts aimed at understanding how and when adaptations improve intervention effectiveness.

### 2.3 CVN Implementation Group

At the outset of the adaptation effort, a 12‐member implementation group was convened, which included members of the research team, members from CVN's central office, the clinical regional director, and the clinic directors at the three identified clinics. The input of this group was critical as they provided access to clinics for recruitment. They also oversee the sites where the intervention will be implemented. This group met biweekly to guide study protocols, support recruitment, and troubleshoot study implementation challenges. The CVN implementation group identified three key informant groups to participate in semi‐structured qualitative interviews, with the goal of gaining a comprehensive understanding of the target population's needs within the clinical context and guiding adaptations to the intervention. These included: (a) CVN administrators and clinicians, to provide insights based on system‐level patterns and direct care experiences, (b) MCM, to share first‐hand perspectives on the lived experiences of military families, and (c) clinicians with prior experience delivering P‐STAIR, to contribute knowledge of the intervention in practice.

### 2.4 Research Team

The research team included members with backgrounds in social work, public health, psychology, and anthropology. We recognize that our disciplinary training and lived experiences influenced how we approached data collection, analysis, and interpretation. Most semi‐structured interviews (*n* = 23) were conducted by the principal investigator and a doctoral research assistant, both trained in clinical social work and community mental health and familiar with the Parenting‐STAIR intervention. A third interviewer, a doctoral‐level anthropologist, conducted two additional interviews. The team reflected diversity across national origin (four countries represented) and racial/ethnic background, which enriched analytic discussions. While no members had personal or family military experience, the PI and senior research assistant had prior experience conducting studies with military‐connected families. The full team met weekly to debrief on study progress, with two qualitative consultants providing training, guidance, and high‐level feedback on methodological rigor and interpretation of findings.

### 2.5 Data Collection

Qualitative consultants and the research team co‐developed three semi‐structured interview guides for: (a) CVN administrators and clinicians, (b) MCM, and (c) clinicians with prior experience delivering P‐STAIR. Guided by Proctor et al.'s implementation outcomes taxonomy (2011) and FRAME (Wiltsey Stirman et al. 2019), topics in the semi‐structured guides for CVN staff and MCM focused on the acceptability, appropriateness, feasibility, and adoption of P‐STAIR. Both guides included questions about the needs and stressors facing this population, gaps in clinical services available to this population at CVN clinics, the relative advantage of P‐STAIR over existing EBIs, and perceived facilitators and barriers to participating in P‐STAIR; however, CVN staff guides included supplemental questions to clarify clinic workflows, while the MCM guides added prompts to elicit personal narratives about their parenting and experiences seeking mental health care. Topics in the semi‐structured guide for clinicians with prior experience delivering P‐STAIR focused on specific components of P‐STAIR and the relative contribution of these components to engagement and desired clinical outcomes. All guides were internally pilot‐tested and modified (e.g., trimmed to include only questions aligned with essential domains; reframed to elicit concrete, unbiased responses) before fielding. The consultants also trained the team in semi‐structured interviewing techniques.

CVN administrators (*n* = 6) and clinicians (*n* = 5), as well as clinicians with prior experience delivering P‐STAIR (*n* = 4), were purposively recruited through the CVN implementation group and the research team's existing networks respectively. These participants completed the informed consent process immediately prior to participating in their interview. MCM (*n* = 10) were recruited via a recruitment flyer placed in CVN clinic waiting rooms and purposively shared by CVN staff with potentially eligible CVN clients. Initially, the research team planned to recruit MCM through the three identified CVN clinics and engage them in focus groups, but recruitment was slower than anticipated. In response, two decisions were made to address recruitment challenges. First, the research team pivoted from focus groups to semi‐structured individual interviews. MCM expressed concerns about privacy so individual interviews supported the gathering of more in depth and potentially more personal information than might have been gathered in a focus group context. Second, CVN central office staff shared study recruitment materials with the entire network of clinics, thereby expanding the pool of potential participants. This yielded two additional participants, which supported us in reaching our recruitment targets. Minimum sample sizes were guided by empirical recommendations (Hennink and Kaiser 2022), the need for sufficient representation—across clinics for staff, and across military‐connectedness, service affiliation, and demographics for mothers—and theoretical saturation (e.g., when participants described similar challenges and support needs, indicating conceptual redundancy).

MCM who were interested in participating scanned a QR code included on the recruitment flyer, which led them to a brief form on which they could indicate their willingness to be contacted by the research team. Upon receiving completed forms, members of the research team called prospective participants, confirmed their eligibility, gathered initial demographic information, completed the informed consent process with each participant, and scheduled them for an interview. MCM were eligible if they were a service member, veteran, or the spouse of a service member or veteran, had at least one child, and were currently or had previously received services at a CVN clinic.

All interviews, which lasted approximately 60 min, took place over HIPAA‐compliant Zoom and were recorded using in‐app functionality. Participants were renamed using an ID number during the interviews. Recordings were uploaded to Sonix, a secure web‐based platform that uses artificial intelligence to convert audio to text, and transcribed verbatim. All transcriptions were then manually checked by a research team member to confirm accuracy and to remove any remaining identifying information. CVN clinicians, including administrators with clinical caseloads, and MCM received US$50 Amazon gift cards for participating in the interviews. Higher‐level administrators, employed by CVN, and clinicians with prior experience delivering the intervention, employed by a separate organization, were not compensated due to their employers' internal policies.

To enhance rigor and trustworthiness, interviewers completed positionality memos before undertaking interviews, as well as memos following each interview, which captured initial thoughts about and observations of each data collection episode. The research team also maintained an audit trail throughout the data collection and analysis phases to track implementation challenges and team decisions. This study was approved by the Institutional Review Board at New York University (FY2024‐8520).

### 2.6 Data Analysis

The present study employed rapid qualitative analysis (RQA)—a rigorous, team‐based approach that produces reliable results comparable to other qualitative methods in a time‐sensitive way—and is supported by prior literature as an effective strategy for adapting interventions (Kowalski et al. 2024; Latimer et al. 2024; Mlunde et al. 2024; Shelley et al. 2022). This iterative process involved several steps to ensure consistency and reliability across the research team. First, the research team developed summary templates for each of the three interview guides. These templates served as a tool to consolidate data, creating an inventory of the critical information from each interview, and were organized by domains. Qualitative consultants reviewed the templates, and multiple team members pilot tested them on a shared transcript to assess usability and consistency in data extraction. Based on this testing, minor revisions were made to improve clarity and ensure summaries yielded comparable results across team members. Once templates were finalized, transcripts were assigned to individual team members for summarization. To support calibration, the research team met weekly to review summaries, address discrepancies in interpretation or level of detail, and ensure a consistent approach to data extraction.

Once summaries were completed, a senior research team member copied the summary template data verbatim into three separate data matrices, one for each of the three interview guides. These structured spreadsheets with columns representing summary template domains and rows corresponding to individual interviews—contain all of the summary data in one place, such that interviews can be easily compared by domain. Using data matrices is considered a valid and rigorous strategy to succinctly condense qualitative data and derive meaning through a comparison of the similarities and differences across transcripts (Abraham et al. 2020; Averill 2002; Hamilton and Finley 2019; Schexnayder et al. 2023).

### 2.7 Adaptation Group

Concurrent with data collection and RQA, a five‐member adaptation group was convened, which included members of the research team and developers of the STAIR and PC‐CARE components of the intervention. Input from this group was critical to consider how conclusions drawn from qualitative data could be meaningfully translated into practical adaptations to the intervention. This group met weekly to review the emergent data and consider adaptations for P‐STAIR. Each member of the adaptation group independently appraised the data matrices to identify patterns and themes and, informed by their subject area expertise, proposed a list of potential adaptations. Guided by FRAME, the adaptation group discussed and unanimously agreed upon an initial set of adaptations aligned with the goal of ensuring the intervention was sensitive to the target population's needs and fit within the clinical context. This consensus list was organized into Table 1.

**TABLE 1:** Documenting Adaptations to the Parenting‐STAIR Intervention using FRAME.

Adaptation to the PSTAIR Intervention	When did the modification occur?	Planned vs. Responsive	Who participated in the decision to modify?	What is modified	For whom/what is the modification made?	What is the nature of the modification?	What was the goal of the modification?
Theme One: Streamlining Treatment to Strengthen Engagement							
Reduce the number of treatment sessions overall	Engagement	Planned; Responsive	Researchers; Intervention Developers; Administrators; Clinicians; MCM	Content	Organization; Target population	Shortening/Condensing	Improve fit; Improve feasibility; Increase engagement
Tailor the manual for efficiency (e.g., offer skills and propose tangible solutions early in treatment).	Pre‐implementation	Responsive	Researchers; Intervention Developers; Administrators; Clinicians; MCM	Content	Target population; Clinicians	Tailoring	Increase engagement; Increase retention; Address cultural factors
Simplify didactics and homework in the manual to ensure that they are brief, manageable, and practicable, acknowledging the many competing and often overwhelming demands faced by MCM	Pre‐implementation	Responsive	Researchers; Intervention Developers; Administrators; Clinicians; MCM	Content	Target population; Clinicians	Tailoring; Shortening/Condensing	Increase engagement; Increase retention; Improve feasibility
Emphasize the importance of establishing and maintaining therapeutic alliance with MCM in the manual and in clinician training.	Pre‐implementation	Responsive	Researchers; Intervention Developers; Administrators; Clinicians; MCM	Content; Training	Target population; Clinicians	Tailoring; Adding elements	Increase engagement; Increase retention; Address cultural factors
Theme Two: Improving Flexibility and Responsiveness							
Leverage a flexible, modular design	Engagement	Planned	Researchers; Intervention Developers	Content	Target population	Tailoring; Spreading	Improve fit; Increase engagement
Add an adherence guide to the manual to track clinician adaptations to session content to better respond to the many competing and often overwhelming demands faced by MCM	Pre‐implementation	Responsive	Researchers; Intervention Developers; Administrators; Clinicians; MCM	Content; Contextual	Target population; Clinicians; Format	Adding elements; Loosening structure	Increase engagement; Increase retention; Address cultural factors
Offer flexibility in treatment delivery method (e.g., in‐person; telehealth; hybrid)	Pre‐implementation	Responsive	Researchers; Administrators; Clinicians; MCM	Contextual	Format	Tailoring	Increase reach; Increase engagement; Increase retention; Address cultural factors
Theme Three: Addressing Stigma and Use of Language							
Tailor the language in recruitment materials and manual to reduce mental health stigma (e.g., use relatable, approachable language to describe trauma, such as distress, overwhelm, and anger; avoid references to PTSD)	Pre‐implementation	Responsive	Researchers; Intervention Developers; Administrators; Clinicians; MCM	Content	Target population	Tailoring	Increase reach; Increase engagement; Address cultural factors; Reduce mental health stigma
Emphasize parenting component in recruitment materials	Pre‐implementation	Responsive	Researchers; Administrators; Clinicians; MCM	Content	Target population	Tailoring	Increase reach; Increase engagement; Reduce mental health stigma
Use inclusive language (e.g., gender‐neutral pronouns; “caregiver” instead of “mother”) in the manual.	Pre‐implementation	Responsive	Researchers; Clinicians; MCM	Content	Target population	Tailoring	Address cultural factors
Broaden inclusion criteria beyond PTSD symptoms	Pre‐implementation	Responsive	Researchers; Intervention Developers	Content	Organization; Target population	Tailoring	Increase reach; Improve fit; Improve feasibility
Theme Four: Integrating Military Contextualized Stressors and Supports							
Add a standalone guide for clinicians that connects the manual to the specific needs (e.g., emotion regulation, self‐care) and stressors (e.g., deployment; relocation) facing the military‐connected population –	Pre‐implementation	Responsive	Researchers; Intervention Developers; Administrators; Clinicians; MCM	Content	Target population; Clinicians	Tailoring; Adding elements	Increase engagement; Address cultural factors
Evaluate examples in the manual to ensure that they address a broad range of trauma exposures (e.g., combat trauma; MST; childhood trauma; secondary trauma)	Pre‐implementation	Responsive	Researchers; Intervention Developers; Administrators; Clinicians; MCM	Content	Target population	Tailoring	Increase engagement; Address cultural factors; Reduce mental health stigma

Abbreviations: FRAME, Framework for Reporting Adaptations and Modifications to Evidence‐based Interventions; MCM, Military‐Connected Mothers; MST, Military Sexual Trauma.

## 3 Results

Demographic information for participants was collected only for MCM. On average, MCM were 35 years old (SD = 6.72) and represented a diverse group of respondents (Table 2). We present key themes that emerged from interviews and informed the adaptation of P‐STAIR. Findings are grouped into four domains: (a) streamlined delivery and engagement, (b) flexibility and responsiveness, (c) stigma and use of language, and (d) military cultural relevance. These themes reflect both shared and contrasting priorities across the adaptation group and key informants involved in the adaptation of this intervention for MCM. Where applicable, we discuss concurrence between different respondent groups and briefly connect these findings to adaptations which are further explicated in the discussion section.

**TABLE 2:** Sample characteristics of Military‐Connected Mothers (*N* = 10).

Characteristics	*M* (SD)/Total number
Age	*M* = 35 (SD = 6.72)
Relationship to the Military	
Active‐Duty Service Member (ADSM)	3
Veteran	3
Spouse of ADSM	3
Spouse of Veteran	1
Military Branch	
Air Force	2
Army	5
Coast Guard	1
Marines	1
Navy	1
Race/Ethnicity	
Black/African American	2
Hispanic/Latina	4
White	4

### 3.1 “The most important thing is the buy‐in”: Streamlining Treatment to Strengthen Engagement

Administrators, clinicians, and MCM all emphasized the importance of initial and ongoing engagement with this population, which manifested in two sub‐themes: (a) reducing burden, and (b) building trust/rapport. With respect to reducing burden, administrators and clinicians stressed the need to improve P‐STAIR's fit with the clinic population and to reduce the time commitment, making it easier for clients to maintain engagement throughout the intervention. Though a subset of clinicians and MCM expressed a desire for longer‐term treatment, all administrators and most clinicians and MCM favored a briefer, more practical approach to reduce burden and ultimately engagement in treatment. Administrators and clinicians described logistical constraints, such as scheduling, heavy caseloads, and a short‐term treatment orientation, which limits the number of sessions that could reasonably be offered in interventions delivered in this context. One administrator said: “[Having a short‐term modality is about] being truly able to identify what we can work on and what will be effective, and then connecting accordingly if [clients] do need longer‐term care, especially with some of these compounded traumas.”

Many MCM echoed this concern regarding time constraints, citing overwhelming competing demands, including caregiving and employment, which limited their availability to participate in mental health services.

I think some moms do sign up for [mental health treatment] and then they have issues … they can't drive somewhere, or their kid gets sick, or, you know, they're, like, having an off‐day. … then they either give up on it or they stop kind of showing up to it. … if the husband is gone or the spouse is gone, they kind of shut down. —MCM; Spouse of service member

Though not explicitly discussed by MCM, administrators and clinicians also identified therapeutic alliance as a critical engagement mechanism. The importance of building trust and offering practical support early in treatment was universally agreed upon among clinic staff, particularly given histories of trauma and mental health stigma facing MCM.

One of the things in my experience as a clinician that's helped with therapeutic alliance is really providing an adequate understanding to the client of the intervention, so that they understand what you're doing and why, and that they … really buy in to the intervention. —Administrator

One clinician further noted: “…There's a lot of distrust. And so, the most important thing would [be] the buy‐in, and them buying into [mental health treatment] being something that's beneficial for them without the suspicion…”.

Adaptations related to this theme focused on reducing the number of sessions and homework between sessions and intentionally focusing on engagement early in treatment through the provision of practical skills and an emphasis on rapport throughout the intervention.

### 3.2 “Shifting on the Axis”: Improving Flexibility and Responsiveness

Administrators, clinicians, and MCM all deemed flexibility essential. In particular, MCM sought a responsive intervention that could be personalized to accommodate their unique set of circumstances, from acute needs to ongoing pressures.

[The] thing that I would like to see changed is not so much a step‐by‐step course outline. ‘This is what it says we have to do, okay.’ [A therapist], you know, who can shift kind of on the axis to kind of help guide in the direction that it needs [to go], not just a baseline out of a binder you're reading. Because that's kind of what a lot of the therapy is like. —MCM; Veteran

Similarly, administrators and clinicians also expressed a need to tailor the intervention while preserving fidelity to core components. This flexibility is crucial in military‐serving settings, where unpredictable family transitions due to deployment or relocation could disrupt the continuity of care.

We've got to be mindful of, like, how long [clients are] going to be in the area … ‘Are you moving?’ Like, ‘is [the new location] going to be, you know, at a clinic within the network where another provider could pick [mental health treatment] right back up?’ Like, what does that look like? … We wouldn't want there to be a disruption in care. —Administrator

Administrators, clinicians, and MCM also expressed interest in flexible delivery modalities, including in‐person, telehealth, and hybrid options, to support accessibility and retention. One Veteran MCM said: “Zoom helps me because … if I had to go in person, I probably wouldn't show up. Even now, there's things, like, I know I need [to do them], but it's hard for me to do them in my own house, much less, like, go somewhere.”

Adaptations related to this theme focused on balancing flexibility in the delivery of P‐STAIR with the need to maintain structured, evidence‐based components through a modular design, consistent tracking of ad‐hoc adaptations, and hybrid treatment delivery through both telehealth and in‐person sessions as needed.

### 3.3 “Some people don't even like the word trauma”: Addressing Stigma

Reducing stigma around mental health and trauma was a central theme expressed among administrators, clinicians, and MCM, who were concerned that diagnostic labels (e.g., PTSD) and clinical language could alienate participants or suppress disclosure due to internalized stigma of mental health, as well as the potential for adverse career implications for service members.

I get a lot of soldiers who say, you know, ‘it's a sign of weakness to ask for help. … we're trained to be soldiers. We're trained to go to war. We're supposed to be strong.’ And so, we get a lot of people who just either ignore the struggles that they're having and the things that they're going through until they, they end up in a crisis … and that spills over to the military family, right? To the children, to the spouses, to the caretakers. —Clinician

Similarly, a MCM, spouse of a service member, said: “Some people don't even like the word trauma because it sounds so scary. … and so maybe saying in a different way or calling it something else because … [people in] the military kind of take on this, like, ‘[I] have to be strong at all times’”.

Though this was not raised by MCM, administrators and clinicians noted that inclusive language should remain a key consideration, reflecting the diversity within the military‐connected population they serve. For example, one administrator said: “It's just kind of the basic components of language implemented. … removing presumptions I think is like the biggest thing.”

Adaptations related to this theme included a focus throughout treatment on using relatable, non‐clinical, and inclusive language, an emphasis on the parenting components of the intervention (over the mental health focus), and a shift in inclusion criteria toward general distress and away from specific clinical diagnoses.

### 3.4 “A lot of trauma doesn't get worked on while in service”: Integrating Military Contextualized Stressors and Supports

Clinicians, administrators, and MCM all discussed the systemic and interpersonal stressors MCM face, including frequent relocations, deployment‐related separations, and loss of social support networks. These experiences were described as compounding traumas that complicated help‐seeking:

[My partner is] always gone, and, like, you want to be upset at them, but then you can't be upset at them because it's their job. … having that, like, past trauma of, like, feeling alone or … powerless—it kind of comes back because you're like, ‘oh, I'm alone. … I can't really do anything about it except try to survive through it.’ —MCM; Spouse of service member

Administrators and clinicians concurred with MCM that discontinuity, detachment, and isolation affect this population's engagement in mental health services.

… if the, the husband is the soldier, …whatever trauma [MCM] have, sometimes it's going to be amplified because they're at home with the kids, and a lot of times they're not leaving the base. They are literally at home with the kid by themselves not making connections with the outside world. … if someone has really been exposed to trauma and they're isolated, the impact on them will be grave. … to me, it seems like they don't get a break. —Clinician

Administrators, clinicians, and MCM, also stressed the importance of addressing exposure to combat trauma, military sexual trauma (MST), and childhood abuse.

I have just had this [trauma] bottled [up from] childhood, and then I'm the first in my family, really that has been at this point with parenting. … I feel alone because I am seeking help, and I do want to do things the right way, because I do want to end that generation of trauma. —MCM; Spouse of veteran

Adaptations related to this theme included the development of a clinician‐facing standalone guide to support awareness of military‐specific needs and attention in the P‐STAIR manual to ensuring examples were inclusive of military‐specific trauma exposures like combat or MST.

## 4 Discussion

In response to the four central themes that emerged from interviews with administrators, clinicians, and MCM, several adaptations were made to P‐STAIR to improve engagement, flexibility, and relevancy to the target population, as well as to address stigma and language concerns. These adaptations are presented below by theme. See also Table 1 for a summary of the intervention adaptations organized by theme.

### 4.1 Streamlining Treatment to Strengthen Engagement

A principal finding was the need to balance the therapeutic content of P‐STAIR with the practical realities MCM face. As a result, two adaptations were made to shorten and condense the treatment protocol. First, the treatment length was reduced from 23 weekly sessions down to between 12 and 16 sessions, depending on modules employed (see below for a discussion of treatment modules). Additionally, homework assignments were simplified in an effort to reduce both the overall time commitment and the complexity of work outside of treatment sessions, while retaining an emphasis on integration of skills into clients' daily lives.

Adaptations also underscored the need for clinicians to be trained in alliance‐building strategies and motivational engagement techniques during P‐STAIR training. Specific prompts were added to the treatment manual to cue clinicians for opportunities to build or support rapport. Further, treatment sessions were reorganized so that tangible skill‐building begins on day one of treatment to promote early and sustained engagement.

### 4.2 Improving Flexibility and Responsiveness

Findings highlighted the importance of flexibility in intervention delivery. To be flexible while still preserving the standardization of an evidence‐based, manualized intervention, P‐STAIR was adapted into PSTAIR‐Modular (PSTAIR‐M), a modular format that allows for variable sequencing and delivery. Modular interventions consist of structured components that can be applied, arranged, or excluded. This method helps ensure consistency and reproducibility of EBIs and is consistent with a growing body of evidence supporting modular interventions for high‐risk and transient populations (Weisz et al. 2012). Modular features also enhance adaptability, allowing PSTAIR‐M to be customized according to client needs and goals. Considering the many intersectional identities held by military‐connected families both within our sample and in this broader population, this modular approach is expected to enable clinicians delivering PSTAIR‐M to more precisely meet the needs of each family.

PSTAIR‐M consists of two modules. Module 1 includes a compressed version of all three phases of treatment offered in the standard 23‐session P‐STAIR. All participants in PSTAIR‐M will receive Module 1, which consists of 11 sessions. Those who do not achieve a sufficient response to treatment after Module 1 as measured by standardized instruments will proceed to Module 2. Three versions of Module 2 were created to offer participants the opportunity to focus more deeply on emotion regulation skill development (Module 2a), on narrative exposure (2b), or on the parent–child relationship (2c). This choice will be guided by standardized assessments and a structured shared decision‐making process between the client and clinician. Each version of Module 2 is composed of one shared decision‐making session, three additional sessions, and a graduation session for a total of between 12 and 16 sessions, depending on module selection.

An adherence guide was also incorporated into the PSTAIR‐M manual to help clinicians document and track intentional modifications offered to clients in‐session, such as crisis management or reintroducing concepts from earlier sessions. Flexibility was also prioritized through offering telehealth and hybrid delivery options to enhance accessibility, particularly for MCM who lack consistent support networks. This aligns with prior research demonstrating that flexible treatment formats improve both reach and retention in trauma‐focused care, particularly for populations facing systemic barriers to participation (Fortney et al. 2015).

### 4.3 Addressing Stigma and Use of Language

Mental health stigma emerged as a significant theme. In an effort to reduce stigma, the manual and recruitment materials were revised to use approachable, everyday language (e.g., “overwhelm,” “distress”), as well as to emphasize parenting challenges—as opposed to mental health—as a primary focus. The manual was revised to use gender‐neutral pronouns and terms such as “caregiver” rather than “mother” to acknowledge diverse family structures and promote welcoming environments for participants who hold different identities.

Additionally, extant research has shown that military culture often reinforces emotional suppression and discourages help‐seeking, particularly among service members and their families (Atuel and Castro 2018; Coll et al. 2011). Therefore, efforts were made to de‐pathologize participants' experiences and validate structural stressors, which were frequently cited as shaping both trauma exposure and service engagement. These adaptations reflect best practices for culturally responsive care and align with previous findings that reframing intervention goals can mitigate stigma and enhance perceived relevance (Rathod et al. 2018).

### 4.4 Integrating Military Contextualized Stressors and Supports

Military cultural relevance was also a salient theme expressed by key informants. In response, the adaptation group developed a clinician‐facing standalone guide to accompany the PSTAIR‐M manual, which is intended to support providers in better understanding military family contexts, stress exposures, and common barriers to care. This approach is in line with PC‐CARE, which has developed supplementary handbooks for tailoring intervention protocols to different diagnostic populations (e.g., children with problematic sexual behaviors; Timmer et al. 2019). Given the focus on attending to varied trauma exposures, the PSTAIR‐M manual was also revised to incorporate content relevant to combat trauma, MST, and adverse childhood experiences. Finally, because administrators, clinicians, and MCM all mentioned that mothers—particularly the spouses of service members and veterans—are often disconnected from their natural support systems and struggle to connect with peers, the research team engaged clinic sites in the development of a resource guide linking MCM to community resources and support groups in their local areas.

## 5 Limitations

The adaptation process focused primarily on pre‐implementation changes; future research is needed to evaluate unplanned or reactive adaptations to the intervention as well as the effectiveness and implementation of PSTAIR‐M in real‐world settings, which will be the focus of a future trial. These implementation outcomes will be systematically measured in an upcoming pilot clinical trial of PSTAIR‐M, tested against treatment as usual among MCM receiving mental health services at CVN clinics. During upcoming phases of this line of research, clinicians using PSTAIR‐M will have an opportunity to provide further feedback on the intervention and MCM who receive PSTAIR‐M will participate in exit interviews which may contribute to further modifications. Further, data were collected exclusively through CVN clinics. Though this approach offered many strengths, this population may be different from those who seek services through more traditional DoD or VA healthcare providers. Additionally, though RQA was an appropriate methodological choice given the goals of the proposed project, this method may not yield the in‐depth findings that could be generated through line‐by‐line coding. Finally, the research team included some members with prior familiarity with P‐STAIR, which may have shaped their interpretation of the data, though inclusion of consultants and study team members without this familiarity was undertaken to mitigate effects of this bias.

This paper describes the systematic adaptation of P‐STAIR, a promising intervention which addresses maternal trauma symptoms and associated parenting skills. The goal of this adaptation process was to ensure the intervention's appropriateness for MCM and for the clinical context in which it would be delivered: community mental health clinics operated by CVN. Guided by qualitative data from administrators, clinicians, and MCM, the adaptation process addressed critical barriers to engagement and retention, considered the need for flexible delivery of EBIs, integrated military cultural relevance, and aimed to reduce stigma while preserving the core components of the intervention. The outcome of this process, PSTAIR‐M, is a briefer, modular intervention we anticipate will be suitable for delivery to MCM.

## Funding

This work was supported by Eunice Kennedy Shriver National Institute of Child Health and Human Development, R21HD112694.

## Conflicts of Interest

The authors declare no conflicts of interest.

## Data Availability

The data that support the findings of this study are available from the corresponding author upon reasonable request. Data will be deposited in a repository upon completion of the project.
